# Child stunting and associated risk factors in selected food-insecure areas in Rwanda: an analytical cross-sectional study

**DOI:** 10.11604/pamj.2022.43.111.35100

**Published:** 2022-10-31

**Authors:** François Niragire, Celestin Ndikumana, Marie Gaudence Nyirahabimana, Cyprien Mugemangango

**Affiliations:** 1 Department of Applied Statistics, University of Rwanda, Kigali, Rwanda,; 2 Department of Governance and Public Administration, University of Rwanda, Kigali, Rwanda,; 3 School of Public Health, Mount Kenya University, Thika, Kenya,; 4 African Centre of Excellence in Data Science, University of Rwanda, Kigali, Rwanda

**Keywords:** Child stunting, poverty, risk factors, logistic regression, Rwanda

## Abstract

**Introduction:**

stunting rates among the under-five children are generally high in Rwanda. They are unexpectedly lower than the national average stunting rate in some districts where poverty rates are the highest in the country. This study aimed to ascertain the key factors that protect children from stunting in these poorest areas, where stunting rates are lower than expected.

**Methods:**

we analysed cross-sectional data from 2019/2020 Rwanda Demographic and Health Survey (RDHS) for 477 under-five children from Karongi, Rulindo, Nyanza, and Gisagara districts. Univariate and bivariate statistical analyses were used to find out the factors to retain for multivariable analysis. We obtained the key risk factors of child stunting through a multivariable binary logistic regression analysis.

**Results:**

the child stunting rate in the study districts was 30 percent, which is three percent lower than the national average rate. Child stunting was negatively associated with a birth weight of at least 2.5 kg (AOR = 0.393, 95% CI: 0.180 - 0.856), a high household economic status (AOR = 0.506, 95%CI: 0.273 - 0.937), urban residence (AOR = 0.467; 95% CI: 0.222 - 0.984), and health insurance coverage (AOR = 0.418; 95% CI: 0.228 -0.767). Children aged one year and above, as well as female-headed households, were associated with at least three times and two times greater odds of child stunting than children aged below 12 months and those from male-headed households respectively.

**Conclusion:**

the nutritional performance of children in the study districts is substantially driven by the high uptake of health insurance, which fosters increased access to healthcare services. To address child-stunting gaps in low-income areas in Rwanda, child nutrition programs should improve the utilization of healthcare services, and leverage the potential high effect of nutrition education, especially during pregnancy and lactation.

## Introduction

Child stunting usually occurs as a failure of linear growth, where the affected children become shorter than expected given their age [1,2]. It adversely affects the physical and mental development of the affected children, and predisposes them to chronic diseases and low productivity in adult life, as well as premature death [1,3,4]. Disparities in the rates of child stunting have been persistently considerable at both global and national levels [2,5]. Globally, stunting affected 149.2 million (22.0%) of children under five years in 2020, declining from 203.6 million (33.1%) in 2000 [2]. Of these stunted children, 84 percent were living in low- and lower- middle-income countries, and 41 percent were living in Africa in 2020 [2]. Although the stunting rates have decreased, Africa is the only place in the world where the number of stunted children increased from 54.4 million (41.5%) in 2000 to 61.4 million (30.7%) in 2020. In particular, the number of stunted children increased from 48.5 million (44.0%) to 55.2 million (32.3%) in sub-Saharan Africa [2]. In Rwanda, the under-five child stunting rate declined from 51% to 33% between 2005 and 2020 [6,7]. However, the national average stunting rate has been persistently higher than the national and global targets [6,8]. In particular, while the world targets an average annual reduction rate (AARR) of about 4.0% [8], Rwanda realised a 15-years AARR of 1.2% between 2005 and 2020 [6,7]. Several studies have investigated the key factors that drive disparities in the child nutrition status between and within different communities [1,9-11]. Within the same community, studies showed that children are not equally affected even when they are fed on the same diet [12]. Generally, existing literature shows that children respond differently to various stunting risk factors, and the latter vary from one community to another [1,4,9,10].

The risk factors for child stunting have been classified into distal, intermediate, and immediate factors [5,13,14]. Often, distal risk factors are subdivided into socioeconomic, demographic, cultural, environmental and development policy-related factors [9,13,15,16]. The immediate risk factors directly influence the child´s access to adequate quality or quantity of food [12]. They include dietary intake and disease [9,14,17]. The intermediate (underlying) factors are those that have direct influence on immediate factors and include those related to infections, access to and availability of food [9,13,17]. These factors are usually observed in terms of limited use or access to health facilities, inadequate care and feeding practices [12,13], and poor hygiene [12,15,16]. The various risk factors are measured at individual child, mother-, household and community levels [13,14,17]. Several studies have shown that individual-level risk factors include the child´s sex, age, and birth weight among other factors [9,10,14,18]. The mother´s level potential risk factors include mother´s age, mother´s education, mother´s height, body mass index, births spacing, antenatal care visits, and maternal nutrition and health seeking behaviours [9-11,14]. The household level factors include household wealth, food security [8,19], household size and household´s environment factors related to hygiene and sanitation [10,11,14,18]. Household headship has also been repeatedly reported as an important factor [20,21]. There is a well-established relationship between child nutrition status and child feeding and breastfeeding practices [12,22]. Following its nutritional benefits, the exclusive breastfeeding during the first six months of life is even recommended for children born to HIV positive mothers [22].

In reality, the risk factors for child stunting are interrelated and complex [1,5,17]. For example, the biophysical environment factors such as elevation, rainfall, temperature, distance to urban areas and market influence household food security and dietary diversity [9]. The household wealth plays a big role in determining its income, main source of water and type of sanitation facilities [23], as well as the level of its access to healthcare services [13]. Poor sanitation and hygiene can lead to diarrhoea and other infections that negatively affect the child growth [15,23]. In general, it is usually expected that children from wealthy or food-secure households experience lower rates of child stunting [19]. Unexpectedly, this relationship between poverty and child stunting rates is no longer obvious in Rwanda. On the one hand, child stunting is widespread in many food-secure districts in the country and, on the other hand, some of the most impoverished districts have child stunting rates that are considerably lower than the national average rate [6,24]. Specifically, poverty rate is above 60% in Nyamasheke district, and it is between 45% and 60% in districts of Burera, Nyabihu, Ngororero, Rutsiro, Nyaruguru, Nyamagabe, Karongi, Rulindo, Nyanza, and Gisagara [24], but child stunting rates are below the national average rate of 33% in Karongi, Rulindo, Nyanza, and Gisagara districts [6]. The Government of Rwanda in collaboration with development partners started several programs and initiatives targeted at improving child nutrition at national scale with a particular focus on the children under age five. In particular, a number of home-grown programs have been implemented including the One Cow per Poor Family programme known as “Girinka”, the Kitchen Garden initiative, locally called “akarima” ´igikoni´, and the Village Kitchen initiative among other programs [25,26]. It has not yet been established whether these programs have reversed the trend of the effect of poverty on child nutritional status. In particular, previous studies have not explored the important within-country exceptions that underlie unexpected reduction in child stunting rates in food-insecure settings [9, 18]. The knowledge of the key protective factors of stunting within the country´s poorest districts would underpin efficient and scalable nutrition intervention programmes. This study aimed to ascertain the key risk factors of child stunting in the poorest districts where child stunting rates are lower than expected in Rwanda.

## Methods

### Study design and data

This is an analytical cross-sectional study. It is based on a quantitative analysis of secondary data from the 2020 Rwanda demographic and health survey (RDHS). The 2020 RDHS was conducted by the National Institute of Statistics of Rwanda (NISR) from 9 November 2019 to 20 July 2020 [6]. The data and permission to use them in this study were obtained from Demography Health Survey (DHS) program. The 2020 RDHS provides nationally representative information on health and demographic indicators including maternal and child health, household environmental, socioeconomic characteristics. In particular, it collected anthropometric data required for the assessment of the nutrition status for children under age five [6].

### Study setting

**Figure 1:**
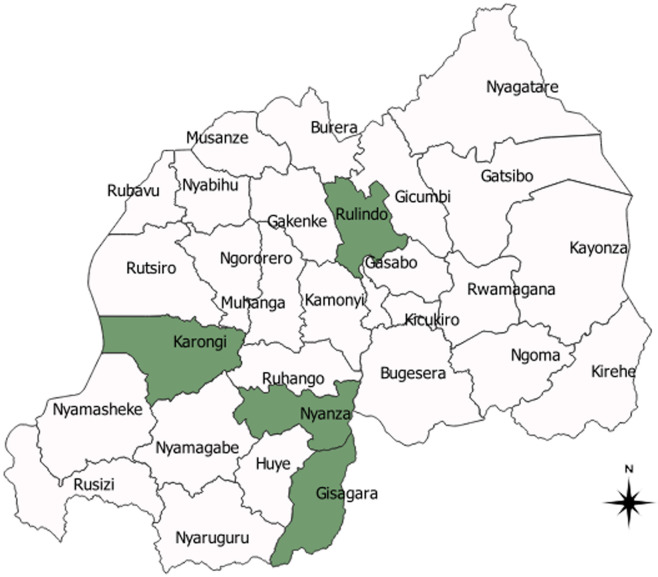
map of Rwanda showing the study districts coloured in green

Rwanda is located in Eastern Africa [7,27]. It is 26,338 square kilometres and has a population of around 12 million [28], and a low human development index (HDI) [29]. In 2017, 38.2% of its population were poor, including 16% who were extremely poor [24]. In the same year, the total poverty rate was 43.1% in rural and 15.8% in urban areas (Figure 1). Rwanda is divided into four administrative provinces and the City of Kigali, which in turn are divided into thirty administrative districts. The present study covered the four poorest districts including Karongi, Rulindo, Nyanza, and Gisagara districts, where child stunting rates were lower than the national average child stunting rate of 33% [6]. Figure 1 shows the four study districts coloured in green. The selected four study districts are predominantly rural and its population mainly practice crop and livestock farming. Poverty rates range from 46.5% in Nyanza to 55.6% in Gisagara [24]. Gisagara and Nyanza are neighbouring districts in the Southern province with population densities of 560 and 481 inhabitants per square kilometre (km) respectively. Rulindo is the most populous (595 inhabitants per square km) while Karongi has the least populous with 334 inhabitants per square km [30]. The main food crops grown in all the study districts include beans and maize. In addition, Rulindo and Karongi grown Irish potatoes, while Nyanza and Gisagara districts also grow rice, sweet potatoes and cassava. Karongi is on the shores of Lake Kivu where silver fish is the main fishery produce.

### Sample design and inclusion criteria

The 2020 RDHS used the fourth Rwanda Population and Housing Census (4RPHC) conducted in 2012 as its sampling frame. A two-stage stratified cluster sampling was used for the 2020 RDHS with a total of 500 census enumeration areas (EAs) selected in the first stage and a systematic sample of 13,000 households selected in the second stage. A full report provides details about the methodology for the 2020 RDHS [6]. This study sample included all livebirths who (i) were under 60 months old by the 2020 RDHS interviews, (ii) were living in the selected four selected districts, namely Karongi, Rulindo, Nyanza, and Gisagara, (iii) whose mothers were interviewed during the 2020 RDHS, and (iv) for whom anthropometric measurements (weight, height) were conducted during the 2020 RDHS. Of the 8092 under five livebirths whose mothers were interviewed during the 2020 RDHS, the present study sample comprised 477 children who met the inclusion criteria for this study.

### Study variables and measurement

**Dependent variable:** the outcome variable for this study was the status of the child´s linear growth. This was categorized as either “stunted” or “normal” linear growth. The values of the outcome variable were coded with 0 (zero) if the child was normal, and with 1 if the child was stunted. With reference to the 2006 WHO Child Growth Standards, stunted children were defined as those whose height-for-age Z-scores fell below minus two standard deviations (-2SD) below the median of the reference population [6,31].

**Independent variables:** the explanatory covariates considered in this study were selected from the 2020 RDHS with reference to the “*UNICEF Conceptual Framework of the analysis of the relationship between poverty, food insecurity, and other underlying and immediate causes of maternal and child undernutrition*” [5]. We also looked at other relevant literature [11,18,32], including the WHO conceptual framework of causes and consequences of child stunting, which was developed from the UNICEF conceptual framework [17].

Based on the reference conceptual framework, the potential factors identified in the 2020 RDHS data include distal, intermediate and proximate factors at the child-, maternal- and household levels. Distal factors comprised household´s economic status, district of residence, type of place of residence, mother´s marital status and religion. The underlying or intermediate factors included child´s environment characteristics including the main source of drinking water, the type of toilet facility used by household members and household size. The underlying maternal and child- level factors included health care services utilization as measured by the number of antenatal care visits during pregnancy, place of delivery, and whether the household was covered by a health insurance scheme. The study also considered socio-demographic intermediate factors including the mother´s age, child birth order, preceding birth interval, mother´s height, mother´s educational level, and mother employment status, child´s sex, and age. Potential immediate factors included in this study are the child´s birth weight (kg), recent diarrheal episodes in 2 weeks preceding the survey interviews, and early initiation of breastfeeding [14]. The immediate factors can be interpreted in terms of adequacy of dietary intake and frequency of disease at individual child level [31]. The conceptual framework assumes that the effect of the distal factors is mediated through the underlying factors to exert their influence on immediate factors, except child´s sex and age, which are non-modifiable factors [5,11,14]. The covariate values or categories for the potential factors are self-explanatory or well documented in the 2020 RDHS report [6] and other published literature [17,33]. The household economic status was derived from the household´s wealth index (quintiles). The households in the ‘poorer’, ‘middle’ and ‘richer’ quintiles were categorized as having a *‘medium’* economic status. Thus, those in ‘poorest’ and ‘richest’ quintiles were categorized as having a ‘low’- and ‘high’- economic status respectively [34]. A child´s weight at birth less than 2.5 kilograms (kg) was classified as “low” [18,35]. Sanitation was measured in terms of whether the household use improved toilet facility, improved source of water. Complete data analysis was performed and all categorical covariates were dummy-coded.

### Statistical analysis

Descriptive statistics were used to generate frequency distributions of the sampled children according to the status of their linear growth (normal or stunted). In the next step, we implemented a series of bivariate analyses with Pearson chi-square tests to identify the individual covariates that had a statistically significant association with the child linear growth status. In the final step, a multivariable logistic regression analysis was conducted to identify the key risk factors of child stunting, the direction and size of their adjusted effects [36]. All the covariates that were significant at a 10% level of significance (p-values < 0.10) in the bivariate analyses were considered for the subsequent multivariable analysis [10,37]. There was no district level data clustering, thus a multivariable fixed effects logistic regression model was considered. Formally, a binary logistic regression modelling of a set of covariates (factors) including the corresponding dummy variables measured on child i is given as equation (1) [36]:

Logit(*p_i_*) = β_0_+ β_1_x_i1_+...+β_κ_x_iκ_


$$\text{logit}\left({\text{p}}_{i}\right)=\ln\left(\frac{{\text{p}}_{i}}{1-{\text{p}}_{i}}\right)$$

where *β* =(*β_0__,_ β_1,…,_ β_k_*) is the vector of linear fixed-effects of the covariates, such that exp(*β_k_*) represents the change in the odds ratio (OR) of the child growth being stunted when the child moves from the reference category to a category of the factor *X_k_* The outcome variable takes on the value 1 with a probability *p*, if the child´s linear growth is stunted, and the value 0 (zero) with a probability 1-*p* otherwise.

The unadjusted odds ratios (OR) and their corresponding 95 percent CIs as well as the adjusted odds ratios (AORs) and their corresponding 95 percent CIs for all the eligible factors were estimated. For each factor the interpretation of results is based on the adjusted odds ratio (AOR) and corresponding 95 percent confidence interval (CI) [36]. These statistics were estimated using IBM SPSS Statistics for Windows, version 23.0.

### Ethics consideration

The protocol for the 2020 RDHS were reviewed and approved by the Rwanda National Ethics Committee, the Institutional Review Board (IRB) of ICF Macro, and the Centres for Disease Control and Prevention (CDC) [6]. Participants provided informed consent of voluntary participation before each survey interview. The DHS program approved our request for the permission to access and use the 2020 RDHS data.

## Results

### Characteristics of the study participants

Among the 477 under-five children sampled, 334 (70.0%) showed a normal linear growth. Thus the stunting rate in the study districts was 30%, including 8.0% who were severely stunted. The frequency distributions for each independent variables were generated according to whether the child growth was stunted or normal. Table 1 shows the frequency distribution of child stunting according to the potential risk factors identified in the 2020 RDHS data. Of all children included in the sample, 127 (26.6%) were residing in Nyanza district, 111 (23.3%) were residing in Gisagara district, 128 (26.8%) were from Karongi, and 111 (23.3%) were from Rulindo district. Stunted linear growth was the most prevalent (44.3%) among children between 24 and 35 months, while normal linear growth rate was the highest (87.0%) in those who were less than 12 months. Majority of the children resided in rural areas (83.6%) and in low-economic status households (50.3%), where the stunting rates were 32.8% and 39.6 % respectively. In total, 73 (30.91%) of the female children and 70 (29.0%) of the male children were nutritionally stunted. An extremely large number of children were born in health facilities (93.7%), and were put to breast within the first hour of birth (89.9%). Of these children, 29.3% and 30.1% were found to be stunted respectively. Similarly, of the 403 (84.5%) children who did not experience any diarrheal disease in the preceding two weeks, 112 (27.8%) were stunted.

**Table 1:** distribution of child stunting and test of its association to potential risk factors (n=477)

Factor	Category	Frequency (%)			p- value
**Stunted (%)**	**Normal (%)**	**Total (%)**
**Child´s sex**	Female	73 (30.9)	163 (69.1)	236 (49.5)	0.653
	Male	70 (29.0)	171 (71.0)	241 (50.5)	
**Child´s age** (months)	Less than 12	13 (13.0)	87 (87.0)	100 (21.0)	<0.001*
	12 - 23	35 (34.3)	67 (65.7)	102 (21.4)	
	24 - 35	39 (44.3)	49 (55.7)	88 (18.4)	
	36 - 59	56 (29.9)	131 (70.1)	187 (39.2)	
**Birth order**	First born	29 (24.0)	92 (76.0)	121 (25.4)	0.095
	Other	114 (32.0)	242 (68.0)	356 (74.6)	
**Preceding birth interval** (years)	First born	29 (24.0)	92 (76.0)	121 (25.4)	0.214
	Less than two	15 (28.8)	37 (71.2)	52 (10.9)	
	Two or above	99 (32.6)	205(67.4)	304 (63.7)	
**Birth weight** (kg)	Below 2.5	18 (50.0)	18 (50.0)	36 (7.5)	0.006*
	2.5 or above	125 (28.3)	316 (71.7)	441 (92.5)	
**Mother´s age** (years)	Below 25	20 (29.9)	47 (70.1)	67 (14.0)	0.988
	25 - 34	74(29.7)	175 (70.3)	249 (52.2)	
	35 - 49	49 (30.4)	112(69. 6)	161 (33.8)	
**Mother´s height** (cm)**	continuous	157.2(5.65)	continuous	477 (100)	0.036*
**Mother´s marital status**	Not in union	35(39.3)	54 (60.7)	89 (18.7)	0.033*
	Living with partner	108 (27.8)	280 (72.2)	388 (81.3)	
**Household size** (persons)	At most five	83 (28.2)	211(71.8)	294 (61.6)	0.291
	Above five	60 (32.8)	123(67.2)	183 (38.4)	
**Sex of household head**	Male	95(25.8)	273 (74.2)	368 (77.1)	<0.001*
	Female	48 (44.0)	61 (56.0)	109 (22.9)	
**Mother´s religion**	Catholic	46 (25.7)	133(74.3)	179 (37.5)	0.254
	Protestant	58 (31.5)	126 (68.5)	184 (38.6)	
	Other	39 (34.2)	75 (65.8)	114 (23.9)	
**Place of residence**	Urban	12(15.4)	66 (84.6)	78 (16.4)	0.002*
	Rural	131 (32.8)	268 (67.2)	399 (83.6)	
**Economic status**	Low	95 (39.6)	145 (60.4)	240 (50.3)	<0.001*
	Medium	24 (27.6)	63 (72.4)	87 (18.2)	
	High	24 (16.0)	126 (84.0)	150 (31.4)	
**Mother´s education**	At most primary	126 (33.0)	256 (67.0)	382 (80.1)	0.004*
	Beyond primary	17(17.9)	78 (82.1)	95 (19.9)	
**Mother is currently employed**	No	19 (22.1)	67 (77.9)	86 (18.0)	0.078
	Yes	124 (31.7)	267 (68.3)	391 (82.0)	
**Type of toilet facility**	Improved facility	80 (28.8)	198 (71.2)	278 (58.3)	0.498
	Others	63 (31.7)	136 (68.3)	199 (41.7)	
**Main source of water**	Improved source	99 (28.6)	247 (71.4)	346 (72.5)	0.290
	Other	44 (33.6)	87 (66.4)	131 (27.5)	
**Antenatal care visits**	No visit	31 (29.8)	73 (70.2)	104 (21.8)	0.467
	One - three	64 (32.8)	131 (67.2)	195 (40.9)	
	Four or above	48 (27.0)	130(73.0)	178 (37.3)	
**Place of delivery**	Health facility	131 (29.3)	316 (70.7)	447 (93.7)	0.216
	Home &Other	12 (40.0)	18 (60.0)	30 (6.3)	
**Put to breast in first hour of birth**	No	14 (29.2)	34 (70.8)	48 (10.1)	0.897
	Yes	129 (30.1)	300 (69.9)	429 (89.9)	
**Had diarrhoea recently**	No	112 (27.8)	291 (72.2)	403 (84.5)	0.015*
	Yes	31 (41.9)	43 (58.1)	74 (15.5)	
**Covered by health insurance**	No	34 (54.8)	28 (45.2)	62 (13.0)	<0.001*
	Yes	109 (26.3)	306 (73.7)	415 (87.0)	
**District**	Nyanza	41 (32.3)	86 (67.7)	127 (26.6)	0.611
	Gisagara	31 (27.9)	80 (72.1)	111 (23.3)	
	Karongi	42 (32.8)	86 (67.2)	128 (26.8)	
	Rulindo	29 (26.1)	82 (73.9)	111 (23.3)	

* the factor is statistically significant,** mean and standard deviation are presented

Children born to mothers who completed at most primary education represented 80.1% of the total sample, and 33.0% of them were stunted. Further, children from households with improved toilet facility and improved main source of water represented 58.3% and 72.5% of the study sample respectively. Of these children, 28.8% and 28.6% were stunted respectively (Table 1). Stunting rate was 44% and 54.8% in households headed by females and those not covered by health insurance respectively. Children from these households represented 22.9 % and 13% of the total sample respectively. Risk factors of under-five child stunting in the study districts In addition to frequency distributions, a series of chi-square tests were performed to test each factor´s association with child´s linear growth status. A single-predictor logistic regression model was fitted for mother´s height (continuous predictor) to test for its association. Table 1 shows the test p-values for each factor. The results showed that 10 factors were statistically associated with child stunting at a 5% level of significance. These include the child age, child birth weight, mother´s height, sex of household head, place of residence, mother´s education level, mother´s marital status, household economic status, episode of diarrheal disease in preceding 2 weeks, and household being covered by a health insurance scheme. In addition, the child birth order, and mother´s current employment status were significantly associated with child stunting at 10% level of significance (p- value < 0.10). Thus, these 12 factors were included in the subsequent multivariable logistic regression analysis (Table 2).

**Table 2:** factors associated with child stunting in the selected districts (n=477)

Factor (reference category)	Unadjusted OR (95% CI)	Adjusted OR (95% CI)
**Child´s age** (Less than 12 months)	1 (reference)	1 (reference)
12 - 23 months	3.496 (1.716 - 7.124)	3.959 (1.840 - 8.519)*
24 - 35 months	5.327 (2.596 - 10.929)	6.065 (2.778 - 13.239)*
36 - 59 months	2.861 (1.476 - 5.543)	3.343 (1.628 - 6.867)*
**Birth weight** (Below 2.5 kg)	1 (reference)	1 (reference)
2.5 or above	0.396 (0.199 - 0.785)	0.393 (0.180 - 0.856)*
**Mother´s height** (continuous)	0.277 (0.077 - 0.996)	0.269 (0.066 - 1.098)
**Mother´s marital status** (Not in union)	1 (reference)	1 (reference)
Living with a partner	0.595 (0.368 - 0.962)	1.012 (0.501 - 2.041)
**Sex of household head** (Male)	1 (reference)	1 (reference)
Female	2.261 (1.450 - 3.527)	2.233 (1.180 - 4.225)*
**Place of residence** (Rural)	1 (reference)	1 (reference)
Urban	0.372 (0.194 - 0.712)	0.467 (0.222 - 0.984)*
**Household economic Status** (Low)	1(reference)	1(reference)
Medium	0.581 (0.340 - 0.994)	0.719 (0.401 - 1.289)
High	0.291 (0.175 - 0.483)	0.506 (0.273 - 0.937)*
**Covered by a health insurance** (No)	1(reference)	1 (reference)
Yes	0.293 (0.170 - 0.506)	0.418 (0.228 -0.767)*
**Mother´s education** (At most primary)	1(reference)	1(reference)
Beyond primary	0.443 (0.251 - 0.780)	0.847 (0.421 - 1.702)
**Had diarrhoea recently** (No)	1 (reference)	1(reference)
Yes	1.873 (1.124 - 3.121)	1.758 (0.971 - 3.184)
**Child´s birth order** (First born)	1(reference)	1(reference)
Other	1.494 (0.931 - 2.398)	1.382 (0.795 - 2.403)
**Mother is currently employed** (No)	1 (reference)	1 (reference)
Yes	1.638 (0.943 - 2.845)	0.918 (0.490 - 1.719)
**Constant**	-	2.110
**Hosmer-Lemeshow test p-value** =**0.641, Cases correctly classified=77.1%**		

* indicates statistically significant Odds ratio

The district of residence was not an important factor (p-value = 0.611) confirming that data were not clustered at district level. The results of the multivariable analysis are presented in Table 2. The results show a Hosmer - Lemeshow test p-value of 0.641, indicating that the model is a good fit to the study data. Further, the logistic regression model classified correctly up to 77.1% of the study subjects. The analytical results in Table 2 also indicates that child age, child´s birth weight, sex of household head, type of place of residence, household cover by a health insurance scheme, household economic status were the statistically significant factors associated with the under-five stunting in the study districts. Specifically, a child´s birth weight of 2.5 kg or above (AOR = 0.393, 95% CI: 0.180 - 0.856), a high household economic status (AOR = 0.506, 95%CI: 0.273 - 0.937), urban residence (AOR = 0.467; 95% CI: 0.222 - 0.984), a household coverage by a health insurance scheme (AOR = 0.418; 95% CI: 0.228 -0.767), and a household male headship (AOR = 2.233; 95% CI: 1.180 - 4.225). Children born with at least 2.5 kg weight were more than 60% less likely to grow stunted compared to those born with a weight below 2.5 kg. The odds of child stunting were 53.3% smaller for children who resided in urban than those in rural areas. Similarly, the odds ratio was about 50% smaller for children who were living in high-economic status households and slightly more than 52% smaller for those who were living in households covered by a health insurance than those who lived in households with low economics status and without health insurance respectively. The likelihood of experiencing a stunted linear growth was more than two times higher for children who were living in female-headed households than those who were living in male-headed households. Compared to children who were less than 12 months old, the odds of a stunted growth increased with the child´s age by at least 3 times for all age groups by the age of 36-59 months. In particular, the odds ratio was almost four times greater for children who were 12 - 23 months (AOR = 3.959; CI: 1.840 - 8.519), more than 6 times greater for those who were 24-35 months (AOR = 6.065; CI: 2.778 - 13.239), and more than three time greater for those who were 36-59 months (AOR = 3.343; CI: 1.162 - 6.867).

## Discussion

The present study investigated the key risk factors of child growth stunting in the selected poorest districts in Rwanda based on the 2019/2020 RDHS data. The average under-five child stunting rate in the studied districts was 30.0%, which is 3% lower than the national average stunting rate of 33%. The residual effect of household wealth remained significant, but it was counterbalanced to a great extent in the study districts. The present study found six key factors that significantly influenced child stunting rate in the study districts: the child´s birth weight, health insurance coverage, place of residence, sex of household head, household´s economic status and child age. Urban residence facilitates utilization and access to markets and hence to diversified food, health care services, and information about child nutrition and healthcare than does the rural residence [9,38]. Health insurance coverage also improves utilization and access to healthcare services and thus the child nutrition outcomes [39]. Child´s birth weight of at least 2.5 kg was a key protective factor of child stunting in the study districts. Several studies have reached to similar finding [9,18]. Further, low birth weight is an important barrier to timing and appropriate breastfeeding as well as utilization of available food [40]. The prevalence of low birth weight in Rwanda was estimated to be 7% of children with known birth weights in 2020 [7]. Children who lived in woman-headed households experienced an increased risk of stunting. Similar finding has been reported by earlier studies [21,41-43]. A woman-headed household is not necessarily poor [20,43], but her workload is increased and can significantly hamper appropriate child feeding and breastfeeding practices. Some other studies did not find any significant child nutrition gaps attributable to household headship [20,43].

The child´s age was negatively associated with child´s normal linear growth. As the child´s age increased the likelihood of growth faltering also increased. It appears that the child´s age is a key determinant for child stunting in Rwanda and in the region [10,17,18]. The results suggest that the effect of the high poverty rates was not completely counterbalanced by all mechanisms in place and that it becomes much stronger as the child feeding becomes more dependent on other food than breast milk. Studies show that stunting can start during pregnancy and its consequences are the most severe when it occurs before the age of two years [1,3,4,27]. In particular case of Rwanda the importance of age factor has triggered a good number of initiatives and health programs that targets to “*prevent stunting in children under two years of age at national scale*” [26]. Child nutrition interventions focus on maternal health care and nutrition, and child breastfeeding and feeding during the first 1000 days (2 years) of child conception and even beyond [4,26]. In the Rwanda context, the attenuated contribution of poverty on child stunting levels in the studied settings can be greatly attributed to a number of home-grown programs that aim to improve the quality of child diet in all households, especially low-income households. Such programs include One Cow per Poor Family programme (“Girinka”), the Kitchen Garden (“akarima” “igikoni”) and the Village Kitchen initiatives [25,26]. These programs promoted the optimal used of locally available food. This study´s strengths stem from the use of population-based data that were collected with standardized tools and methods [7]. In addition, there were a rigorous application of adequate sample design and statistical methods. This study´s limitations are attached to the general nature of cross-sectional survey data. The latter cannot establish causal relationships, but only associations between variables. Further, the RDHS mainly collected demographic and health characteristics, and hence information related to agricultural policy, food markets, and household-level food security indicators were not available in the study data. However, these limitations do not affect the quality of the study results because they have no bearing on the quality of data and the performance of the data analysis methods.

## Conclusion

The present study identified the key protective factors of the under-five child stunting in the selected four poorest districts in Rwanda. These are dominated by demographic and socio-economic factors. The results suggest that the effect of high poverty rates in the study districts was largely offset by the effect of a high uptake of health insurance, which is known to increase the utilization of healthcare services. The latter, together with the household´s economic status considerably explain the observed high prevalence of normal birth weight and, ultimately, normal linear growth of the children living in the study districts. Child nutrition programs that target the poor settings in Rwanda and other similar low-income countries should improve access to and utilization of healthcare services, and leverage the potential high effect of nutrition education, especially during pregnancy and lactation.

### What is known about this topic

- Chronic undernutrition among the under-five has remained at the centre of development challenges in Rwanda for decades;
- Multiple initiatives have led to mixed results and unevenly affected the usual nutrition trends defined by poverty levels.

### What this study adds

- Demographic and socioeconomic factors dominate key risk factors of child stunting in poor areas in Rwanda;
- Improved access to healthcare services through a high uptake of health insurance, which contributed significantly to improvement in child nutrition outcomes in food-insecure settings.

## References

[1] Roediger R, Hendrixson DT, Manary MJ (2020). A roadmap to reduce stunting. Am J Clin Nutr.

[2] United Nations Children´s Fund, World Health Organization, World Bank Group (2021). Levels and trends in child malnutrition: key findings of the 2021 Edition of the Joint Child Malnutrition Estimates.

[3] Victora CG, Adair L, Fall C, Hallal PC, Reynaldo M, Richter L (2008). Maternal and child undernutrition: consequences for adult health and human capital. Lancet.

[4] Dewey K, Begum K (2011). Long-term consequences of stunting in early life. Maternal & Child Nutrition.

[5] United Nations Children´s Fund (2013). Improving Child Nutrition: The achievable imperative for global progress.

[6] National Institute of Statistics of Rwanda, Ministry of Health (MOH) [Rwanda], ICF (2021). Rwanda Demographic and Health Survey 2019-20 Final Report. Kigali, Rwanda and Rockville.

[7] National Institute of Statistics of Rwanda, Ministry of Health (MOH) [Rwanda], ICF International (2016). Rwanda Demographic and Health Survey 2014-15.

[8] United Nations (2014). Transforming our World: The 2030 Agenda for Sustainable Development.

[9] Weatherspoon DD, Miller S, Ngabitsinze JC, Weatherspoon LJ, Oehmke JF (2019). Stunting, food security, markets and food policy in Rwanda. BMC Public Health.

[10] Nkurunziza S, Meessen B, Van geertruyden J-P, Korachais C (2017). Determinants of stunting and severe stunting among Burundian children aged 6-23 months: evidence from a national cross-sectional household survey, 2014. BMC Pediatr.

[11] Fenske N, Burns J, Hothorn T, Rehfuess EA (2013). Understanding Child Stunting in India: A Comprehensive Analysis of Socio-Economic, Nutritional and Environmental Determinants Using Additive Quantile Regression. PLoS One.

[12] United Nations Children´s Fund (2021). Fed to Fail? The Crisis of Children´s Diets in Early Life 2021. Child Nutrition Report.

[13] Stewart CP, Iannotti L, Dewey KG, Michaelsen KF, Onyango AW (2013). Contextualising complementary feeding in a broader framework for stunting prevention. Maternal & Child Nutrition.

[14] Boah M, Azupogo F, Amporfro DA, Abada LA (2019). The epidemiology of undernutrition and its determinants in children under five years in Ghana. PLoS One.

[15] Cumming O, Cairncross S (2016). Can water, sanitation and hygiene help eliminate stunting? Current evidence and policy implications. Matern Child Nutr.

[16] Vilcins D, Sly PD, Jagals P (2018). Environmental Risk Factors Associated with Child Stunting: A Systematic Review of the Literature. Ann Glob Health.

[17] Wirth JP, Rohner F, Petry N, Onyango AW, Matji J, Bailes A (2017). Assessment of the WHO Stunting Framework using Ethiopia as a case study. Matern Child Nutr.

[18] Nshimyiryo A, Hedt-Gauthier B, Mutaganzwa C, Kirk CM, Beck K, Ndayisaba A (2019). Risk factors for stunting among children under five years: a cross-sectional population-based study in Rwanda using the 2015 Demographic and Health Survey. BMC Public Health.

[19] Petrou S, Kupek E (2010). Poverty and childhood undernutrition in developing countries: A multi-national cohort study. Soc Sci Med.

[20] Staten LK, Dufour DL, Reina JC, Spurr GB (1998). Household headship and nutritional status: Female-headed versus male/dual-headed households. American Journal of Human Biology.

[21] Haidar J, Kogi-Makau W (2009). Gender differences in the household-headship and nutritional status of pre-school children. East African Medical Journal.

[22] World Health Organization (2009). Infant and young child feeding: model chapter for textbooks for medical students and allied health professionals.

[23] Freeman MC, Garn JV, Sclar GD, Boisson S, Medlicott K, Alexander KT (2017). The impact of sanitation on infectious disease and nutritional status: A systematic review and meta-analysis. Int J Hyg Environ Health.

[24] National Institute of Statistics of Rwanda (2018). Rwanda Poverty Profile Report: The Fifth Integrated Household Living Conditions Survey (EICV 5) 2016/17.

[25] Lovell G Reducing Child Stunting with Village Kitchens 2019.

[26] Ministry of Local Government, Ministry of Health, Ministry of Agriculture and Animal Resources (2014). Rwanda National Food and Nutrition Policy. Kigali.

[27] United Nations Children´s Fund (UNICEF), World Health Organization (WHO), World Bank Group (2020). Levels and trends in child malnutrition: key findings of the 2020 Edition of the Joint Child Malnutrition Estimates.

[28] National Institute of Statistics of Rwanda, Ministry of Finance and Economic Planning (2014). Rwanda Fourth Population and Housing Census 2012. Thematic Report: Population Projections.

[29] UNDP (2019). Human Development Report 2019.

[30] National Institute of Statistics of Rwanda (NISR), MINECOFIN (2014). Rwanda Fourth Population and Housing Census 2012.

[31] World Health Organization (WHO) (2006). WHO Child Growth Standards, Length/height-for-age, weight-for-age, weight-for-length, weight-for-height and body mass index-for-age: Methods and development.

[32] Tasic H, Akseer N, Gebreyesus SH, Ataullahjan A, Brar S, Confreda E (2020). Drivers of stunting reduction in Ethiopia: a country case study. Am J Clin Nutr.

[33] Kandala NB, Madungu T, Emina J, Nzita K, Cappuccio F (2011). Malnutrition among children under the age of five in the Democratic Republic of Congo (DRC): does geographic location matter?. BMC Public Health.

[34] Niragire F, Achia TNO, Lyambabaje A, Ntaganira J (2017). Child mortality inequalities across Rwanda districts: a geoadditive continuous-time survival analysis. Geospat health.

[35] Abubakari A, Kynast-Wolf G, Jahn A (2015). Prevalence of abnormal birth weight and related factors in Northern region, Ghana. BMC Pregnancy Childbirth.

[36] Stoltzfus JC (2011). Logistic Regression: A Brief Primer. Acad Emerg Med.

[37] Stahel WA (2021). New relevance and significance measures to replace p-values. PLoS One.

[38] Fox K, Heaton TB (2021). Child nutritional status by rural/urban residence: a cross-national analysis. Journal of Rural Health.

[39] Lu C, Mejía-Guevara I, Hill K, Farmer P, Subramanian SV, Binagwaho A (2016). Community-Based Health Financing and Child Stunting in Rural Rwanda. Am J Public Health.

[40] Zoleko-Manego R, Mischlinger J, Dejon-Agobé JC, Basra A, Mackanga JR, Akerey Diop D (2021). Birth weight, growth, nutritional status and mortality of infants from Lambaréné and Fougamou in Gabon in their first year of life. PLoS One.

[41] Onyango A, Tucker K, Eisemon T (1994). Household headship and child nutrition: a case study in western Kenya. Social science & medicine.

[42] Chindime C (2007). Household headship and nutritional status of toddlers. African Population Studies.

[43] Wendt A, Hellwig F, Saad GE, Faye C, Mokomane Z, Boerma T (2021). Are children in female-headed households at a disadvantage? An analysis of immunization coverage and stunting prevalence: in 95 low- and middle-income countries. SSM - population health.

